# Transformed Visual Working Memory Representations in Human Occipitotemporal and Posterior Parietal Cortices

**DOI:** 10.1523/ENEURO.0162-25.2025

**Published:** 2025-07-08

**Authors:** Yaoda Xu

**Affiliations:** Department of Psychology, Yale University, New Haven, Connecticut 06510

**Keywords:** fMRI, occipitotemporal cortex, posterior parietal cortex, vision, visual object representation, visual short-term memory, visual working memory

## Abstract

Recent fMRI studies reported transformed representations between perception and visual working memory (VWM) in the human early visual cortex (EVC). This is inconsistent with the still widely cited original proposal of the sensory account of VWM, which argues for a shared perception-VWM representation based on successful cross-decoding of the two representations. Although cross-decoding was usually lower than within-VWM decoding and consistent with transformed VWM representations, this has been attributed to experimental differences between perceptual and VWM tasks: once they are equated, the same representation is expected to exist in both. Including human participants of both sexes, this study compared target and distractor representations during the same VWM delay period for the same objects, thereby equating experimental differences. Even with strong VWM representations present throughout the occipitotemporal cortex (OTC, including EVC) and posterior parietal cortex (PPC), fMRI cross-decoding revealed significant representational differences between distractors (perception) and targets (VWM) in both regions. Similar differences existed between target encoding (perception) and delay (VWM), being greater in OTC than PPC, indicating more invariant target representations in PPC than OTC. As only part of the sensory input is usually task-relevant, sustaining sensory input in VWM without selection/refinement/consolidation is both taxing and unnecessary. Transformed representations, mediated by task goals and associative areas coding task-relevant information (e.g., PPC), can easily account for these and other recent findings. A task-driven transformed account of VWM thus better captures the nature of VWM representation in the human brain (including EVC) than the sensory representations originally proposed by the sensory account of VWM.

## Significance Statement

The original proposal of the sensory account of visual working memory (VWM) argues for a shared representation between perception and VWM in sensory areas. This assumption, however, was not thoroughly tested due to differences in experimental settings in prior studies. Using fMRI cross-decoding and closely matched experimental conditions, this study compared object representations when they were VWM targets and distractors and during the encoding and delay period of VWM. Both comparisons revealed significant representational differences between perception and VWM in human sensory areas. These results are inconsistent with the sensory nature of VWM representations as it was originally proposed. Instead, they support a task-driven transformed account of VWM in which sensory input is selected/refined/consolidated before VWM storage in these areas.

## Introduction

Prior research has put forward the sensory account of visual working memory (VWM) representation in the human brain (D’Esposito and Postle, 2015; Serences, 2016; Christophel et al., 2017), stating that “the systems and representations engaged to perceive information can also contribute to the short-term retention of that information” (p. 118; D’Esposito and Postle, 2015). There are thus two critical components of this account: (1) the involvement of sensory regions in VWM and (2) a shared representation between perception and VWM in sensory regions. Regarding the second component, Serences et al. (2009) stated that “the pattern of delay activity was qualitatively similar to that observed during the discrimination of sensory stimuli, suggesting that WM representations in V1 are reasonable “copies” of those evoked during pure sensory processing”(p. 207). Similarly, D’Esposito and Postle (2015) stated that sensory information is retained in working memory in a highly stimulus-specific manner “most parsimoniously explained as the persistent activation of the sensory representations themselves” (p. 119). By sustaining perceptual representations in sensory regions in VWM when stimuli are no longer in view, the sensory account attempts to provide a mechanistic understanding of VWM. Although these two particular studies were published ten or more years ago, they are still highly cited: A quick Google Scholar search shows that both papers have been cited 283 and 928 times since 2021, 103 and 306 times since 2024, and 17 and 70 times since 2025, respectively. Thus, these studies are still considered highly relevant in today’s working memory research.

While the existence of VWM signal in sensory regions has been well established (Harrison and Tong, 2009; Serences et al., 2009; Bettencourt and Xu, 2016a; Rademaker et al., 2019), whether perception and VWM share the same representation, however, is less clear. For one thing, it is at odds with goal-directed visual information processing. At any given moment, only part of the sensory input is task-relevant. Sustaining the sensory representation without selection and refinement is both unnecessary and taxing, especially given our limited information processing capacity (Xu, 2018a). Indeed, significant transformations between perception and VWM have been reported in recent studies, inconsistent with the sensory account of VWM. For example, Kwak and Curtis (2022) reported in VWM the retention of abstracted perceptual representation of the original perceptual input in the human early visual cortex (EVC; see also Duan and Curtis, 2024, and Yan et al., 2023). Li and Curtis (2023) further showed that the transformed EVC VWM representation can guide subsequent behavior. Xu (2023) showed that the content of VWM in the human occipitotemporal cortex (OTC) is more similar to the information encoded and maintained in the human posterior parietal cortex (PPC), a goal-directed adaptive visual processing region (Xu, 2028a,b), than to the sensory information initially encoded into OTC.

One key initial evidence supporting the sensory account of VWM comes from cross-decoding, whereby a linear classifier trained to distinguish neural representations in a separate perceptual task can successfully do so for the representations held in VWM (Harrison and Tong, 2009; Albers et al., 2013; Rademaker et al., 2019). Notably, cross-decoding performance, although significant, is usually lower than that of within-decoding, in which the classifier is trained and tested within VWM. Such a cross-decoding drop has been attributed to experimental differences since sensory and VWM data were typically collected in different fMRI runs under different task environments and attentional engagements. For example, in the sensory task of Harrison and Tong (2009), participants identified centrally presented letters while ignoring low-contrast grating flashing in the surround, while in the VWM task, they encoded the centrally presented high-contrast grating. Experimental differences could thus easily contribute to the observed cross-decoding drop without representations necessarily being different between perception and VWM. If perception and VWM indeed share a representation, then when experimental settings are properly controlled for, very little cross-decoding drop should be expected. Meanwhile, a significant cross-decoding drop can also signal a representational transformation between perception and VWM, consistent with recent reports. Thus, a critical test of the sensory nature of VWM comes down to whether or not a cross-decoding drop is still present when the testing condition is properly matched. This present study aims to differentiate between these two possibilities by reanalyzing data from a previous study with well-matched experimental settings for perception and VWM.

## Materials and Methods

The present study reanalyzed the data from a recently published study (Xu, 2024) in which the same objects could appear either as VWM targets or distractors during the delay period in different trials. By training a linear classifier on a pair of objects when they were distractors during the delay period to then decode the same objects when they were VWM targets during the delay period, the present study examined if there was still a cross-decoding drop compared with when training and testing were both performed on VWM targets during the delay period. Here, because both types of training and testing occurred during the delay period, the experimental setting was well-matched. Moreover, given that representations were much stronger for the distractors (visible and sensory) than for VWM targets (invisible) in the sensory regions, a sensory account would predict a much greater VWM decoding when the classifier was trained on the distractors than on the VWM targets. If, however, a significant cross-decoding drop or chance-level cross-decoding was obtained, it would provide strong evidence showing distinct sensory and VWM representations and argue against the sensory nature of VWM representations.

Experimental details of the present study have been reported extensively in the original publication (Xu, 2024). They are reproduced/summarized here for the readers’ convenience. New analysis details pertinent to the present study are described in detail.

### Participants

Fourteen (nine females) healthy human participants with normal or corrected to normal visual acuity, all right-handed and aged between 18 and 35 years, took part in the two scanning sessions of the study. All participants gave their informed consent prior to the study and received payment for participation. The study was approved by the Committee on the Use of Human Subjects at Yale University. Three additional participants also took part in the study but did not complete the second of the two scanning sessions. Their data were not analyzed.

### Main VWM experiment

**Figure 1.**
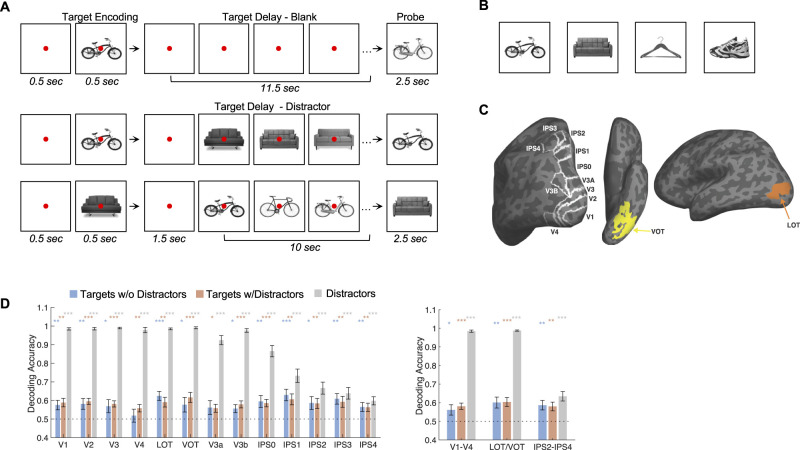
Experimental design and example brain regions of interest (ROIs). ***A***, Example trials showing the trial sequence. In each trial, a single target is shown; after an extended delay period filled with either a blank screen or a sequential presentation of exemplars of another object, a probe is shown. The probe was either an exact match or a different exemplar of the same object type. The entire image set is available from the online data deposit. ***B***, The four types of objects used. ***C***, Example ROIs shown on the inflated cortical surfaces. Reproduced from Vaziri-Pashkam and Xu (2019). ***D***, Decoding accuracies during the VWM delay period for targets in trials without and with distractors and for distractors. The colored symbols above the bars mark the decoding significance of each bar compared with chance (0.5). Error bars indicate SEM. *p < 0.05, **0.01 < *p* < 0.001, ***p < 0.001.

This experiment used four types of objects, namely, bikes, couches, hangers, and shoes (sneakers; Fig. 1*B*). To increase task difficulty and ensure that a visual code was employed to retain VWM representation, similar-looking exemplars of a given object were used, and the probe object at the end of the VWM delay was either the same target object or another exemplar from the same object (see https://osf.io/8rbkh/ for the complete set of images used). All images were placed on a white square (subtended 9.73° × 9.73°) and shown on a larger gray background. Each VWM trial contained a central presentation of a target object, a prolonged blank delay, and a probe object (Fig. 1*A*). The probe object was either an exact match to the target image or a different exemplar of the same type of object. Each trial was 15 s long, with the timing of the different events as follows: fixation (0.5 s) in the form of a looming red dot to alert the participants to the imminent presentation of the target images, target image (0.5 s), blank delay with a red fixation dot (1.5 s), blank delay or distractor delay with a red fixation dot (10 s), and probe image (2.5 s). In trials with a distractor delay, 20 distractor images were shown, with the 10 unique exemplars from the distractor object each shown twice with no back-to-back repetition of the same exemplar. Each distractor image was shown for 0.3 s, followed by a 0.2 s blank screen before the next distractor image was shown. There were a total of 16 unique trials in each run, including 12 trials for all the target and distractor object combinations (four target objects multiplied by three distractor objects) and 4 trials in which no distractors were shown. Each run started and ended with an 8 s blank period with a blue fixation dot. Successive VWM trials were sandwiched by a blank period with a blue fixation dot. Of the 15 such intertrial blank periods, 3 were 8 s long, and 12 were 2 s long, and they were randomly distributed. In a first scan session, 13 or 14 runs of data were collected from each participant, with each run lasting 5 min and 4 s; in a second scan session, 14 runs of data were collected from each participant.

The design of the present study was modeled closely after Harrison and Tong (2009), who asked participants to remember different exemplars from two orientation categories (i.e., with exemplars varying ±3 or ±6° from 25 or 115°). Responses in Harrison and Tong (2009) were then averaged over exemplars within each orientation category, and decoding was performed at the category level (25° vs 115°). Following this design, in the present study, target decoding was performed at the object level (e.g., bikes vs couches) by including all trials containing exemplars from the same object in the same condition (e.g., all trials with bike exemplars were treated as bike trials); likewise, distractor decoding was also performed at the object level when the aggregated distractor responses from the delay period were used for distractor decoding. Thus, although targets were remembered at the stimulus level, which requires the encoding of both object- and exemplar-specific information, only the basic-level object information contributed to VWM decoding. Similarly, when participants viewed the distractors and noticed the differences among the distractor exemplars, they also encoded both object- and exemplar-specific information from the distractors; however, only the basic-level object information contributed to distractor decoding from the aggregated distractor response during the delay period. In this regard, target and distractor decoding was well matched, and both involved the decoding of basic-level object information rather than exemplar-specific information.

### Localizer experiments

#### Topographic visual regions

These regions were mapped with flashing checkerboards using standard techniques (Sereno et al., 1995; Swisher et al., 2007) with parameters optimized following Swisher et al. (2007) to reveal maps in the parietal cortex. Specifically, a polar angle wedge with an arc of 72° swept across the entire screen (19.07° × 13.54° of visual angle). The wedge had a sweep period of 32 s, flashed at 4 Hz, and swept for eight cycles in each run (for more details, see Swisher et al., 2007). Participants completed four to six runs, each lasting 4 min and 36 s. All participants were asked to detect a dimming that could occur anywhere within the polar angle wedge, thereby ensuring attention to the whole wedge.

#### Lateral and ventral occipitotemporal regions

To identify these ROIs, following Kourtzi and Kanwisher (2000) and as we have done previously (Jeong and Xu, 2017; Vaziri-Pashkam and Xu, 2017, 2019; Vaziri-Pashkam et al., 2019; Taylor and Xu, 2022), participants viewed blocks of objects and scrambled objects (all subtended approximately 9.73° × 9.73°). The images were photographs of gray-scaled common objects (e.g., cars, tools, and chairs) and phase-scrambled versions of these objects. Participants monitored a slight spatial jitter which occurred randomly once in every 10 images. Each run contained four blocks of each of the objects, phase-scrambled objects, and two other conditions that were used to define another brain region. Each block lasted 16 s and contained 20 unique images, with each appearing for 750 ms and followed by a 50 ms blank display. Besides the stimulus blocks, 8 s fixation blocks were included at the beginning, middle, and end of each run. Each participant was tested with two runs, each lasting 4 min and 40 s.

### MRI method

Each participant completed two experimental sessions (1.5 h) and a localizer session (1.5 h) containing topographic mapping and functional localizers. MRI data were collected using a Siemens Prisma 3T scanner, with a 32-channel receiver array head coil. Participants lay on their backs inside the scanner and viewed the backprojected display through an angled mirror mounted inside the headcoil. The display was projected using an LCD projector at a refresh rate of 60 Hz and a spatial resolution of 1,280 × 1,024. An Apple MacBook Pro laptop was used to create the stimuli and collect the motor responses. Stimuli were created using MATLAB and Psychtoolbox (Brainard, 1997).

A high-resolution T1-weighted structural image (0.8 mm × 0.8 mm × 0.8 mm) was obtained from each participant for surface reconstruction. All blood oxygen level-dependent data were collected via a T2*-weighted echoplanar imaging pulse sequence that employed multiband RF pulses and simultaneous multislice (SMS) acquisition. For both the main experiment and the localizers, 72 axial slices (2 mm isotropic), 0 skip, covering the entire brain were collected (TR, 800 ms; TE, 37 ms; flip angle, 52°; SMS factor, 8).

### Data analyses

fMRI data were analyzed using FreeSurfer (surfer.nmr.mgh.harvard.edu), FsFast (Dale et al., 1999), and in-house MATLAB codes. LIBSVM software (Chang and Lin, 2011) was used for the MVPA support vector machine analysis. fMRI data preprocessing included 3D motion correction and linear and quadratic trend removal. After reconstructing the inflated 3D cortical surface of each participant using the high-resolution anatomical data, we projected the fMRI data from that participant onto their native cortical surface. As was done in a recent study (Xu, 2023), all fMRI responses were analyzed directly on the inflated cortical surface (vertices) rather than on the cortical volume (voxels) of each participant, including ROI definition and the main VWM analysis, as surface-based analysis has been shown to exhibit more sensitivity and better spatial selectivity (Oosterhof et al., 2011; Brodoehl et al., 2020).

#### ROI definitions

Following the detailed procedures described in Swisher et al. (2007) and as was done in our prior publications (Bettencourt and Xu, 2013, 2016a,b; Vaziri-Pashkam and Xu, 2017, 2019; Vaziri-Pashkam et al., 2019), by examining phase reversals in the polar angle maps, we identified areas V1 to V4, V3a, V3b, and IPS0 to IPS4 in each participant (Fig. 1*C*). Following Kourtzi and Kanwisher (2000) and as was done in our prior studies (Jeong and Xu, 2017; Vaziri-Pashkam and Xu, 2017, 2019; Vaziri-Pashkam et al., 2019; Taylor and Xu, 2022, 2024), LOT and VOT were then defined as a cluster of continuous voxels in the lateral and ventral occipital cortex, respectively, that responded more to the intact than to the scrambled object images (Fig. 1*C*). LOT and VOT loosely correspond to the location of LO and pFs (Malach et al., 1995; Grill-Spector et al., 1998; Kourtzi and Kanwisher, 2000) but extending further into the temporal cortex in an effort to capture the continuous activations often seen extending into the ventral temporal cortex.

#### VWM decoding analysis

With the length of our VWM trials being 15 s, for each surface vertex, we estimated the fMRI response amplitude at each TR from the onset of the trial up to 24 s, totaling 30 TRs (with each TR being 800 ms). This was done separately for the trials in each of the 16 conditions. To obtain these estimates, we first constructed 30 finite impulse response functions (FIRs) corresponding to each TR of each condition's trials. As each condition appeared only once in a run, given the short intertrial interval (mostly 2 s) and the lag in hemodynamic responses, it was not possible to accurately estimate the amplitudes of the FIRs in each run. To obtain reliable amplitude estimates for the FIRs, following the procedure developed in a recent study (Xu, 2023), we combined five or six runs together, as detailed below, before applying GLM modeling to derive the beta weight estimate for each of the 480 FIR functions (30 TRs multiplied by 16 categories). Because the trial onset times were jittered with respect to TR onsets, trial onset times were rounded to the nearest TR before GLM modeling. To obtain independent training and testing data for pattern decoding, we split the runs into odd and even halves in each of the two scan sessions, with each split containing six or seven runs depending on the total number of runs acquired in a given session. We then applied a GLM to each six-run combination if the split contained seven runs and to each five-run combination plus a combination including all six runs if the split contained six runs. This resulted in six beta weight estimates for each FIR function in each split for each surface vertex. The beta weights of all the vertices in a given ROI formed our fMRI response pattern for that ROI. For each ROI and across the four data splits, we thus had a total of 28 patterns for each TR and each condition. Note that within a split, the seven patterns were not independent of each other as they were estimated from shared runs; however, the patterns between the different splits came from independent runs and were thus independent of each other.

To generate a response amplitude time course from each ROI for each condition, we averaged all the beta weights across all the surface vertices within an ROI and from all 28 patterns. Based on the peak responses from all the ROIs and conditions, we defined an encoding period (from 4 to 6.4 s) and a delay period (from 9.6 to 12 s before the onset of the probe). We then averaged the four beta weights within each period to generate an average response for each of these two VWM process stages.

Prior to our decoding analysis, to remove amplitude differences across categories, ROIs, and VWM processing stages, following our previous studies (Bettencourt and Xu, 2016a; Vaziri-Pashkam and Xu, 2017, 2019; Vaziri-Pashkam et al., 2019; Xu, 2023), we *z*-normalized each fMRI response patterns. For a given ROI and for a pair of conditions, we used SVM for pattern decoding (LIBSVM; Chang and Lin, 2011). We trained a decoder using all the response patterns from three splits of the data (totaling 21 patterns) to test its performance on the left-out data split (7 patterns). Training and testing were thus done on independent data sets. We rotated the training and testing order, with each data split serving as the test split and the remaining three as the training splits, and averaged the results from all four rotations.

Training and testing were performed separately for each pair of conditions of interest and the decoding results were averaged across all relevant pairs of conditions to derive the average decoding performance for a given analysis. To directly compare the different ROIs, maximize the contrast, and streamline the analysis, based on the anatomical locations, we formed three ROI sectors at the three ends of the ROIs and averaged the decoding performance within the ROIs in each sector: a posterior sector including lower visual areas V1–V4, a ventral sector including object areas LOT and VOT, and a dorsal sector including higher PPC areas IPS2–IPS4. Within each ROI sector, we performed decoding within each ROI and then averaged the results, rather than forming a combined ROI containing the individual ROIs and then performing decoding. This was done to allow equal weighing of the results from the different ROIs: as size differed across the different ROIs and different participants, decoding based on a combined ROI may bias the results toward the larger ROIs, which could further differ across participants. Our main comparisons focused on the differences among the three ROI sectors. We performed two types of decoding.

**Figure 2.**
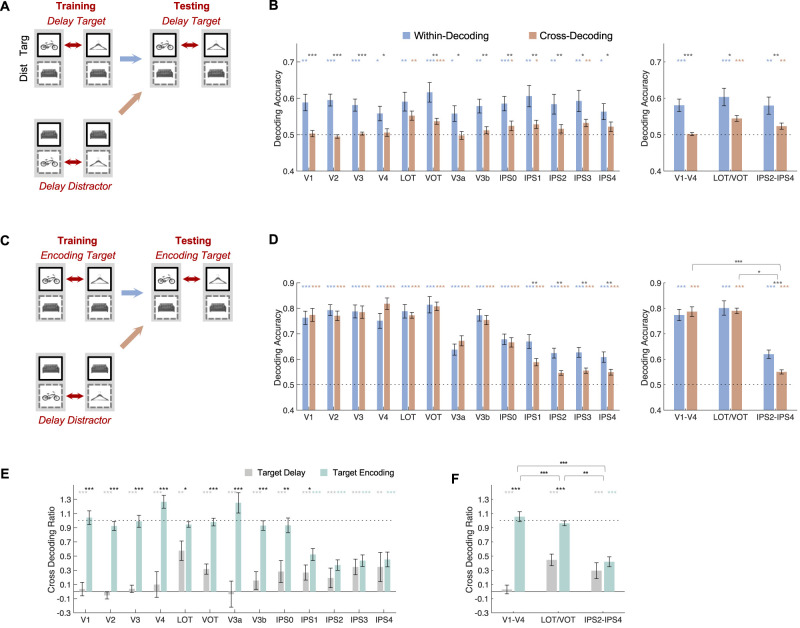
Cross-distractor decoding of VWM targets for trials with distractors. ***A***, An illustration of within-target and cross-distractor target decoding during the delay period. A classifier was either trained by VWM targets or by distractors to decode VWM targets. The distractors during VWM target training and the targets during distractor training were matched (e.g., couches in the examples shown). See Extended Data Figure 2-1 for additional results showing that the same results were obtained regardless of whether or not the irrelevant object remained the same or different in distractor training and target testing. ***B***, Results of ***A*** for all the ROIs (left) and the three ROI sectors (right). ***C***, Same as ***A***, except target training and decoding occurred during the encoding period. Distractor training still occurred during the delay period. ***D***, Results of ***C*** for all the ROIs (left) and the three ROI sectors (right). ***E***, Cross-decoding ratios for both types of cross-decoding for all the ROIs. ***F***, The same results as in ***E*** for the three ROI sectors. In ***B*** and ***D***, the colored symbols above the bars mark the decoding significance of each bar compared with chance (0.5). The black symbols right above the colored symbols mark the significance of decoding drop between within and cross-decoding. In ***E*** and ***F***, the colored symbols above the bars mark the significance of the ratio compared with 1. The black symbols right above the colored symbols mark the significance of the ratio difference between the two types of cross-decoding. In ***B***, ***D***, and ***F***, the black symbols above the brackets mark the significance of the difference in either cross-decoding drop (***B*** and ***D***) or ratio for the two types of cross-decoding (***F***) between pairs of ROI sectors. Error bars indicate SEM. *p < 0.05, **0.01 < *p* < 0.001, ***p < 0.001.

**Figure 3.**
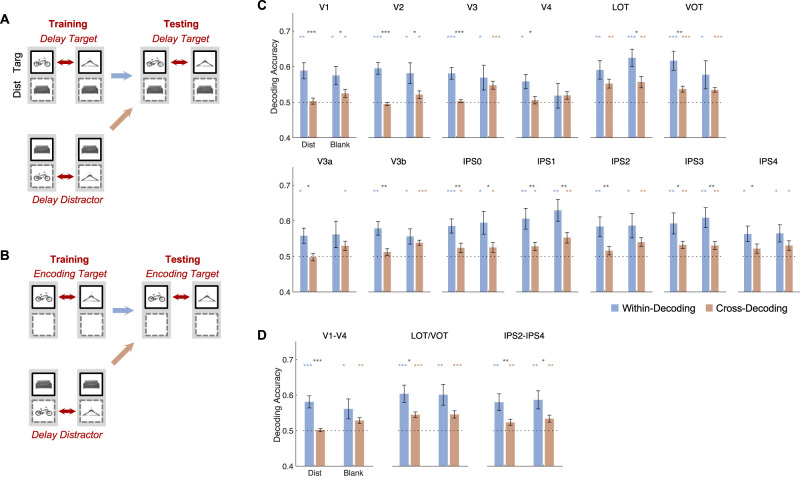
Comparing cross-distractor decoding of VWM targets for trials with and without distractors. ***A***, An illustration of within-target and cross-distractor target decoding during the delay period for trials with distractors (same as in Fig. 2*A*). ***B***, Same as ***A***, but for trials without distractors. ***C***, Results of ***A*** and ***B***, plotted for separately each ROI. ***D***, Same as ***C***, for the three ROI sectors. In ***C*** and ***D***, the colored symbols above the bars mark the decoding significance of each bar compared with chance (0.5). The black symbols above the colored symbols mark the significance of the decoding drop between within and cross-decoding. Error bars indicate SEM. *p < 0.05, **0.01 < *p* < 0.001, ***p < 0.001.

(1) Cross-decoding between distractors and targets. Here, as illustrated in Figure 2*A*, we trained a classifier to decode a pair of objects when they were distractors during the delay period to decode the same pair of objects when they were targets during the delay period. The irrelevant object in both cases was the same (e.g., training to classify A vs B in the training data when they were distractors with C being the target in both to then decode A vs B in the testing data when they were targets with C being the distractor in both). We then compared this cross-decoding with within-decoding in which training and testing were done within the target objects (e.g., training on A vs B in the training data when they were targets with C being the distractor in both to decode A vs B in the testing data when they were targets with C being the distractors in both). The results are shown in Figure 2*B*. The results did not differ if the irrelevant object differed between training and testing (e.g., training on A vs B when they were distractors with C being the target in both to decode A vs B when they were targets with D being the distractor in both; Extended Data Fig. 2-1). In addition to decoding targets from trials with distractors during the delay period, the distractor-trained classifier was also asked to decode targets from these trials during the encoding period (Fig. 2*C*,*D*) and targets from trials without distractors during the delay period (Fig. 3).

10.1523/ENEURO.0162-25.2025.f2-1 Figure 2-1 Training distractors to decode targets in trials with distractors. **A.** The irrelevant object was the same across training and test (e.g., training on A vs. B when they were distractors with C being the target in both to decode A vs. B when they were targets with C being the distractor in both). **B.** The irrelevant object differed across training and test (e.g., training on A vs. B when they were distractors with C being the target in both to decode A vs. B when they were targets with D being the distractor in both). Error bars indicate s.e. * *p* < .05, ** .01 < *p* < .001, *** *p* < .001. Download Figure 2-1, TIF file.

Note that the cross-decoding between distractors and targets was performed only in one direction (from distractors to targets) rather than both ways, which would include training targets to decode distractors. This was done due to the ceiling and highly saturated distractor decoding performance, especially from sensory regions (Fig. 1*D*). In other words, distractor decoding performance no longer tracked the underlying signal strength. As such, performing decoding both ways could significantly distort the results. Instead, a more conservative approach was taken here to use the distractor-trained decoder to decode targets. If perception and VWM share the same representation, cross-decoding should be similar to within-decoding (which was trained and tested on VWM targets), if not greater, as the distractor-trained classifier would be more robust than the VWM-trained classifier given the much stronger distractor than VWM representation during the delay period, especially in sensory areas. However, if cross-decoding is substantially lower than within-decoding or at chance, it would provide strong evidence indicating very little representational overlap between perception and VWM.

Although we could also train on the targets during the delay to decode the distractors (cross-target distractor decoding), it is unclear whether this type of decoding would be informative. The key question here is what we should compare this type of decoding with to draw valid conclusions. If we compare it with within-target decoding during the delay period (i.e., train on targets to decode targets), then because the distractor signal is ultrastrong, we may get high distractor decoding due to this, even when there is only partial overlap between target and distractor representations. This would prevent us from comparing distractor and target decoding to draw firm conclusions regarding whether targets and distractors share representations during VWM delay. If we compare cross-target distractor decoding with within-distractor decoding (i.e., train on distractors to decode distractors), because the classifier is weaker when trained with the targets than with the distractors during delay, a drop in performance in cross-target distractor decoding compared with within-distractor decoding could be due to that, rather than a difference in target and distractor representations. Again, we will not be able to draw firm conclusions here. It is thus unclear whether obtaining cross-target distractor decoding would be informative here.

To examine the differences between within- and cross-decoding and to account for decoding performance differences across the different ROIs and the different decoding periods, following prior studies documenting tolerance of visual object representations in OTC to changes such as position and size (Xu and Vaziri-Pashkam, 2022) and documenting VWM representation across different distractor conditions (Xu, 2024), the within- and cross-decoding measures were combined to form a cross-decoding ratio for the two types of decoding. This was done by first subtracting 0.5 from the within- and cross-decoding accuracies and then taking the ratio of the two resulting values. A ratio of 1 would indicate equally good decoding performance within VWM and across distractors and VWM and a complete generalization of perception and VWM, whereas a ratio of 0 would indicate a complete failure of such generalization. The results are shown in Figure 2, *E* and *F*.

**Figure 4.**
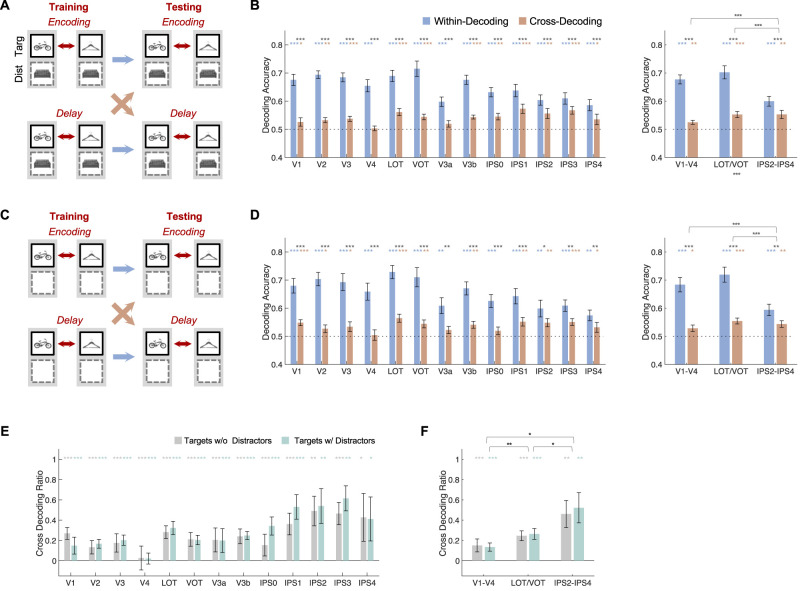
Within and cross-decoding between VWM encoding and delay periods. ***A***, An illustration of within and cross-decoding between encoding and delay periods for trials with distractors. ***B***, Results of ***A*** for all the ROIs (left) and the three ROI sectors (right). ***C***, An illustration of within and cross-decoding between encoding and delay periods for trials without distractors. ***D***, Results of ***C*** for all the ROIs (left) and the three ROI sectors (right). ***E***, Cross-decoding ratios for both types of trials for all the ROIs. ***F***, The same results for the three ROI sectors. In ***B*** and ***D***, the colored symbols above the bars mark the decoding significance of each bar compared with chance (0.5). The black symbols right above the colored symbols mark the significance of decoding drop between within and cross-decoding. In ***E*** and ***F***, the colored symbols above the lines/bars mark the significance of the ratio compared with 1. The black symbols right above the colored symbols mark the significance of the ratio difference between the two types of trials. In ***B***, ***D***, and ***F***, the black symbols above the brackets mark the significance of the difference in either cross-decoding drop (***B*** and ***D***) or overall ratio (***F***) between pairs of ROI sectors. Error bars indicate SEM. *p < 0.05, **0.01 < *p* < 0.001, ***p < 0.001.

(2) Cross-decoding between VWM encoding and delay for targets. In this analysis, a classifier was trained to decode a pair of targets during either VWM encoding or delay, and its performance was then tested during both VWM encoding and delay. The results were averaged across both directions of training and testing (since the within-decoding performance was not saturated/at the ceiling for either). This was done separately for trials with and without distractors (Fig. 4*A*,*C*). The results are shown in Figure 4, *B* and *D*.

As in (1), to evaluate potential differences in the two types of trials and to account for decoding performance differences across the different ROIs and decoding periods, the within and cross-decoding measures were combined by computing a cross-decoding ratio for the two types of trials following the procedures described in (1). The results are shown in Figure 4, *E* and *F*.

It is worth noting that pairwise decoding was conducted for all the analyses here. Since four types of targets and distractors were used in the present study, one might wonder if a single four-way decoder should be used. A four-way decoder, unfortunately, would not work for the present study. Although there were four types of target objects A, B, C, and D, and the same four types of distractor objects, given that target and distractor objects differed in a given trial, the four types of distractor objects did not appear equally often for each type of the target objects (i.e., target A with distractors B, C, and D, but never with A; target B with distractors A, C, and D, but never with B; so on and so forth). Thus, when performing a four-way target decoding, differences among the distractor objects among the four types of target objects would necessarily contribute to the results, and the decoding results would reflect differences among the distractors (given their much stronger representation) than the targets. This would significantly distort the findings. This problem, however, could be avoided with pairwise decoding. Here, the distractor objects were kept constant when performing target decoding, e.g., decode A versus B when C was the distractor for both. Thus, it was essential to perform pairwise decoding in the present study.

#### Statistical analyses

*t* tests were used to assess within and cross-decoding performance against chance (one sample, one-tailed, as only effects above chance were meaningful), cross-decoding drop (paired, one-tailed, as the effect was expected to be either null or with cross-decoding being lower than within-decoding), and pairwise comparisons among the three ROI sectors (paired, two-tailed). Correction for multiple comparisons was applied using the Benjamini–Hochberg method (Benjamini and Hochberg, 1995) for the number of tests of the same type performed within each ROI or sector and for the three pairs of tests performed across the three ROI sectors. Repeated measures ANOVAs were used to assess main effects and interactions between variables of interest.

## Results

In this study, human participants retained real-world objects in VWM, either with or without distraction during the delay period, with the same objects serving as either targets or distractors in different trials (Fig. 1*A*,*B*). The target and distractor objects came from the same four types of objects sharing roughly the same outline (i.e., bikes, couches, hangers, and shoes). To increase task difficulty and ensure that a visual code was employed to retain VWM representation, similar-looking exemplars of a given object shown in the same viewpoint were used, and the probe object at the end of the VWM delay was either the same target object or another exemplar from the same object.

As in a previous study (Bettencourt and Xu, 2016a), behavioral performance did not differ between trials with and without distractors (Xu, 2024). This may not be surprising. Given the ubiquitous presence of distractions in everyday life (Ongchoco and Xu, 2024), for VWM to be useful, it needs to be fairly robust to distraction. Indeed, as reviewed by Xu (2017), the effect of distraction was either absent or very small; when it was present, it was in studies when very precise sensory representations were retained—which is not how information is typically stored and used in VWM in everyday life (e.g., we rarely have to encode the precise object color or orientation in the real world). Thus, it should come as no surprise that VWM is fairly robust to distraction and that no effect of distraction on behavioral performance was found in the present study.

The main analysis of the present study focused on the fMRI responses extracted from regions of interest (ROIs) across OTC and PPC (Fig. 1*C*), including early visual areas V1 to V4, object processing regions in lateral occipitotemporal (LOT) and ventral occipitotemporal (VOT) cortex, and parietal topographic areas V3a, V3b, and IPS0 to IPS4. As in a recent study (Xu, 2023), fMRI responses were analyzed directly on the inflated cortical surface (vertices) rather than on the cortical volume (voxels) of each participant, as surface-based analysis has been shown to exhibit more sensitivity and better spatial selectivity (Oosterhof et al., 2011; Brodoehl et al., 2020). From the averaged fMRI response time courses of each ROI, encoding and delay periods were defined, fMRI responses were averaged within each period, and fMRI response patterns were generated for each ROI and each condition (see Materials and Methods). Significant VWM decoding was obtained across almost all the ROIs regardless of distractor presence, and decoding did not differ between trials with and without distractors in all the ROIs (Fig. 1*D*; see also Xu, 2024). To compare across the ROIs, maximize the contrast, and streamline the analysis, three sectors at the three ends of the ROIs were created, and the decoding performance was averaged within the ROIs in each sector: a posterior sector including lower visual areas V1–V4, a ventral sector including object areas LOT and VOT, and a dorsal sector including higher PPC areas IPS2 to IPS4. Comparison across sectors revealed that the strengths of VWM representations were similar across the three ROI sectors and across trials with and without distractors (Xu, 2024). As in Bettencourt and Xu (2016a) and Rademaker et al. (2019), successful decoding was also obtained for distractors during the delay period in all the ROIs (Fig. 1*D*; see also Xu, 2024).

### Transformation between VWM target and distractor representations

In the present study, because the same objects appeared as both VWM targets and distractors during the delay in different trials, there was a unique opportunity to test the extent to which perception and VWM shared the same representation under the same experimental setting. To accomplish this, a classifier was trained to decode a pair of objects when they were distractors, and its ability to decode the same objects was then tested when these objects were VWM targets during the delay period (Fig. 2*A*). For example, a classifier would be trained to differentiate a bike and a hanger when they were distractors in trials with couches being the VWM targets. The classifier would then be asked to differentiate a bike and a hanger when they were VWM targets with couches being the distractors. This was then compared with decoding within VWM (e.g., classifying a bike and a hanger target when couches were the distractors during both training and testing). If perception and VWM share the same representation, cross-decoding should be similar to within-decoding, if not greater, as the distractor-trained classifier would be more robust than the VWM-trained classifier given the much stronger distractor than VWM representation during the delay period, especially in sensory areas (Fig. 1*D*). However, if cross-decoding is substantially lower than within-decoding or at chance, it would indicate very little representational overlap between perception and VWM.

While cross-decoding from distractors to VWM targets during the delay period was greater than chance in LOT/VOT and IPS2–IPS4, interestingly, it was no greater than chance in V1–V4 (Fig. 2*B*; see the asterisk marking the significance level; corrected for the two comparisons made in each ROI sector). Comparison between within- and cross-decoding revealed a significant drop across all the ROI sectors (Fig. 2*B*; the same results were obtained when the irrelevant object differed between training and testing; Extended Data Fig. 2-1). Comparisons across the sectors using repeated measures ANOVA revealed a main effect of cross-decoding drop and a main effect of sector (*F*’s > 5.78, *p*’s < 0.0083), but no interaction between the two (*F*_(2,26)_ = 1.03, *p* = 0.37). Pairwise comparisons among the sectors revealed that the amount of the cross-decoding drop did not differ across any pairs of ROI sectors (*t*’s < 1.30, *p*’s > 0.43; two-tailed and corrected for the three comparisons made). There were thus significant representational transformations between perception and VWM across all three ROI sectors. While there was still significant overlap between perceptual and VWM representations in LOT/VOT and IPS2–IPS4, such overlap appeared to be absent in V1–V4.

If the cross-decoding drop observed above was a result of information transformation between perception and VWM, then when the distractor-trained classifier was to ask to decode the perceptual representations of the targets during VWM encoding instead of delay, no cross-decoding drop should be present in the sensory areas (Fig. 2*C*). Indeed, significant cross-decoding was found in all the ROI sectors, and critically, no cross-decoding drop was present in V1–V4 or LOT/VOT compared with when training and testing were performed within the targets during encoding (Fig. 2*D*). In sensory regions (i.e., V1–V4 and LOT/VOT), target representations during VWM encoding and distractor representations during VWM delay thus shared similar representations. Meanwhile, a significant cross-decoding drop was still present in IPS2–IPS4, indicating a difference in representation. Repeated measures ANOVA revealed that, across the sectors, there was no main effect of cross-decoding drop (*F*_(1,13)_ = 1.49, *p* = 0.24), a main effect of sector, and an interaction between the two (*F*’s > 11.08, *p*’s < 0.001). Further pairwise comparisons revealed that the cross-decoding drop was greater in IPS2–IPS4 than in either V1–V4 or LOT/VOT (*t*’s > 2.83, *p*’s < 0.022; two-tailed and corrected); the drop did not differ between LOT/VOT and V1–V4 (*t*_(13)_ = 1.67, *p* = 0.12; two-tailed and corrected).

Note that although the baseline decoding performance was higher in OTC than in PPC in the above analysis, simulations from a prior study showed that decoding accuracy followed the underlying signal strength in a linear manner (except when decoding was very close to chance 0.5 or very close to 1; Xu, 2024). This enabled a direct comparison of cross-decoding drops even when the baseline decoding strengths differed. Simulations further showed that a cross-decoding drop was absent when two conditions merely differed in SNR, resulting in different baseline decoding performances, but otherwise shared the same underlying representation (Xu and Chun, 2025). The cross-decoding results reported here thus reflected the nature of the underlying visual representation rather than artifacts of the decoding measure used.

To directly compare cross-decoding during VWM delay and encoding, to streamline the analysis, and to account for decoding performance differences across the different ROIs, following prior studies documenting tolerance of visual object representations in OTC to changes such as position and size (Xu and Vaziri-Pashkam, 2022) and tolerance of VWM representation across different distractor conditions (Xu, 2024), the within- and cross-decoding measures were combined to form a cross-decoding ratio for decoding during VWM encoding and delay. This was done by first subtracting 0.5 from the within- and cross-decoding accuracies and then taking the ratio of the two resulting values. A ratio of 1 would indicate equally good decoding performance within VWM and across distractors and a complete generalization of perception and VWM, whereas a ratio of 0 would instead indicate a complete failure of such generalization. This analysis yielded similar results across the individual ROI and the three ROI sectors (Fig. 2*E*,*F*). Comparing the ratios across the sectors using repeated measures ANOVA revealed a main effect of cross-decoding type (VWM encoding vs delay), a main effect of sector, and an interaction between the two (*F*’s > 11.99, *p*’s < 0.001). Further pairwise comparisons revealed that the ratio difference between the two types of cross-decoding was smaller in IPS2–IPS4 than in either V1–V4 or LOT/VOT and smaller in LOT/VOT than in V1–V4 (*t*’s > 3.68, *p*’s < 0.01; two-tailed and corrected).

Thus, even under an identical experimental setting, there existed a large drop in cross-decoding between perception (i.e., distractors) and VWM across all the ROIs, showing that in neither OTC nor PPC, perception and VWM shared the same representation. Notably, while some representational overlap existed between perception and VWM in LOT/VOT and IPS2–IPS4 (i.e., with above-chance cross-decoding), such overlap was not observed in EVC (i.e., with chance-level cross-decoding). As expected, distractor representations were highly similar to target representations during target encoding in OTC, indicating a shared perceptual representation for both. However, distractor and target representations differed even during encoding in higher PPC regions, showing that these regions represent the same perceptual input differently based on the goal of visual processing. This is consistent with the goal-driven and adaptative nature of visual information processing in PPC (for extended reviews, see Xu, 2018a,b; see also Jeong and Xu, 2013; Xu and Jeong, 2015; Bracci et al., 2017; Vaziri-Pashkam and Xu, 2017).

### The impact of distraction on VWM representation transformation

The preceding analysis showed that in trials with distractors during the delay period, even when the experimental testing conditions were matched, there were significant differences between VWM and perceptual representations across OTC and PPC. Given that distractions are ubiquitous in everyday life, these results suggest that in real-world vision, VWM, and perceptual representations likely differ substantially. Nevertheless, it may be argued that the representational difference between VWM and perception could be a result of distraction, with the presence of distractors altering target representation. Xu (2024) showed that targets and distractors formed orthogonal representations in PPC. Such a representational geometry allowed targets to be read out independently of distraction and effectively resisted distraction in PPC. The same representational scheme, however, was absent in OTC, with target representations being different in different distractor conditions. It is thus possible that, in the absence of distraction, similar VWM and perceptual representations may still be obtained.

To test this idea, in this analysis, after a classifier was trained on a pair of objects when they were distractors, the classifier was tested on the same pair of objects when they were targets both in trials with distractors (as in the previous analysis) and in trials without distractors (Fig. 3*A*,*B*), and the results were compared. The results from each ROI were reported in Figure 3*C*. Significant cross-decoding drop for trials without distractors was still present in a number of ventral sensory areas, including V1, V2, and LOT (although the results from the other ventral areas were noisy, they largely showed the same trend). When a repeated measures ANOVA was performed in each ROI with trial type (with vs without distractors) and decoding (within vs cross-decoding) being the independent variables, a majority of them showed a main effect of cross-decoding drop; critically, none of the ventral sensory areas showed an interaction between trial type and cross-decoding (Table 1). Confirming these results, the three ROI sectors all showed a main effect of cross-decoding but no interaction effects (Table 2; Fig. 3*D*).

**Table 1.** Repeated measures ANOVA results for the individual ROIs for trial type (trials with vs without distractors), decoding (within vs cross-decoding), and their interaction

ROI	Trial type (*F*/*p*)	Decoding (*F*/*p*)	Interaction (*F*/*p*)	ROI	Trial type (*F*/*p*)	Decoding (*F*/*p*)	Interaction (*F*/*p*)
**V1**	0.05/0.83	17.81/0.001	1.64/0.22	**V3a**	1.73/0.21	3.14/0.099	0.38/0.55
**V2**	0.15/0.70	19.44/<0.001	1.87/0.19	**V3b**	0.03/0.87	4.47/0.054	6.20/0.027
**V3**	0.87/0.37	4.69/0.049	3.51/0.083	**IPS0**	0.12/0.74	9.18/0.0097	0.048/0.83
**V4**	0.43/0.52	1.19/0.30	2.36/0.15	**IPS1**	2.91/0.11	10.47/0.0065	<0.001/0.98
**LOT**	2.18/0.16	4.53/0.053	1.08/0.32	**IPS2**	1.92/0.19	4.53/0.053	0.72/0.41
**VOT**	1.97/0.18	4.25/0.059	1.16/0.30	**IPS3**	0.27/0.61	7.78/0.015	0.87/0.37
				**IPS4**	0.12/0.74	6.52/0.024	0.11/0.75

**Table 2.** Repeated measures ANOVA results for the three ROI sectors for trial type (trials with vs without distractors, decoding (within vs cross-decoding), and their interaction

ROI	Trial type (*F*/*p*)	Decoding (*F*/*p*)	Interaction (*F*/*p*)
**V1–V4**	0.05/0.82	8.63/0.012	3.24/0.095
**LOT/VOT**	0.0062/0.94	5.22/0.040	0.019/0.89
**IPS2–IPS4**	1.14/0.30	7.74/0.016	0.082/0.78

Because cross-decoding involved training the classifier on the strong sensory representations of the distractors to decode the much weaker VWM representations, cross-decoding could be high as long as there was some overlap in representation. This worked against finding a cross-decoding drop when within-decoding involved training and testing within the weak VWM signal. Even so, a significant cross-decoding drop was still seen across a number of ventral sensory areas in trials without distractors. Moreover, despite the cross-decoding drop being numerically smaller for trials without than those with distractors, the cross-decoding drop did not significantly differ across the two trial types in the ventral sensory areas. Overall, these results showed the presence of a significant representational transformation between perception and VWM in ventral sensory areas even when distractors were not shown during the delay period.

### Transformation between VWM encoding and delay representations

Results from the first analysis showed that, in OTC, a substantial amount of transformation in visual representations occurred between perception and VWM. However, the amount of transformation in PPC remained unknown as distractor and target representations differed during both encoding and delay. Although PPC could contain similar representations during VWM encoding and delay, a transformation could also occur as incoming sensory information was consolidated into VWM representations. To evaluate these two possibilities, in this analysis, a classifier was trained to decode a pair of targets during encoding; it was then asked to decode the same pair of targets during both encoding (within-decoding) or delay (cross-decoding). The same analysis was also carried out in the reverse direction, with training performed during delay and testing performed during both encoding and delay. The results were averaged across both directions of training and testing (Fig. 4*A*,*C*). Note that although cross-decoding between VWM encoding and delay has been performed in previous studies (Riggall and Postle, 2012; Emrich et al., 2013; Sreenivasan et al., 2014 ; Kwak and Curtis, 2022; Xu, 2023), none of these studies included trials with distractors; additionally, some of them either did not include PPC areas or did not explicitly quantified the difference between OTC and PPC areas.

For both trials with distractors (Fig. 4*A*) and trials without distractors (Fig. 4*C*), significantly above-chance cross-decoding was found in all the ROI sectors; however, there was also a significant cross-decoding drop across all the ROI sectors, with the drop being greater in OTC than in PPC areas (Fig. 4*B*,*D*; see also results for the individual ROIs in these figures). Direct comparison across the ROI sectors using repeated measures ANOVA revealed a main effect of cross-decoding drop, a main effect of sector, and an interaction between the two (*F*’s > 13.15, *p*’s < 0.001) for both trial types. Further pairwise comparisons revealed that the drop in cross-decoding was much smaller in IPS2–IPS4 than in either V1–V4 or LOT/VOT (*t*’s > 4.62, *p*’s < 0.001), with no difference between the latter two (*t*’s < 0.25, *p*’s > 0.40) for both trial types.

Similar results were also obtained using the cross-decoding ratio measure (as detailed in the first analysis) for both the individual ROIs and the three ROI sectors (Fig. 4*E*,*F*). Specifically, comparisons across the sectors revealed no effect of trial type (*F*_(1,13)_ = 0.13, *p* = 0.73), an effect of sector (*F*_(2,26)_ = 5.30, *p* = 0.012), and no interaction between the two (*F*_(2,26)_ = 0.37, *p* = 0.69). Further pairwise comparisons revealed that the overall cross-decoding ratio was higher in IPS2–IPS4 than in either V1–V4 or LOT/VOT and higher in LOT/VOT than in V1–V4 (*t*’s > 1.79, *p*’s < 0.048).

Overall, these results revealed significant cross-decoding between VWM encoding and delay. These results by themselves, however, did not necessarily indicate representational overlap between VWM encoding and delay. Due to the sluggishness of the fMRI signal, partial fMRI signal overlap between encoding and delay could also result in successful cross-decoding between these two stages of VWM processing. A significant cross-decoding drop, however, did signal representational transformation between VWM encoding and delay in both OTC and PPC. Given that substantially less transformation was found in PPC than in OTC, even with transformation, object representations appeared to be more stable in PPC than in OTC across the two stages of VWM processing. Notably, these effects were not impacted by the presence of distractors during the delay period, showing that the change in representation was not a result of distraction in VWM.

## Discussion

A host of recent studies reported visual representation transformations between perception and VWM in human EVC (Kwak and Curtis, 2022; Li and Curtis, 2023; Xu, 2023; Yan et al., 2023; Duan and Curtis, 2024; see also behavioral evidence from Harrison and Bays, 2018; Yörük et al., 2020). These results are at odds with one aspect of the sensory account of VWM storage as it was originally proposed, which argues that representations formed during perception are maintained during VWM in sensory regions (D’Esposito and Postle, 2015; Serences, 2016; Christophel et al., 2017). One key evidence supporting this account was the finding that a linear classifier trained to distinguish neural representations in a separate perceptual task could do so successfully for the representations held in VWM (Harrison and Tong, 2009; Albers et al., 2013; Rademaker et al., 2019). Notably, cross-decoding performance was usually lower than within-decoding when the classifier was trained and tested within VWM. This cross-decoding drop, although consistent with there being a representational transformation between perception and VWM, has been attributed to differences in the experimental settings and stimuli between perceptual and VWM tasks, with the assumption being that should these factors be properly controlled for, minimal cross-decoding drop is expected (Harrison and Tong, 2009). In light of recent evidence arguing against sensory representations in VWM, it is critical to reexamine this initial evidence and document whether a cross-decoding drop is still present when experiment conditions are properly matched between perception and VWM. Doing so would clarify an important aspect of the original proposal of the sensory account of VWM, which is still widely cited today, and improve our understanding of the nature of VWM representations.

The present study analyzed the data from a recent study (Xu, 2024) in which the same objects appeared either as VWM targets or distractors during the delay period in different trials, thereby creating the same experimental setting for decoding perceptual and VWM representations for the same set of objects. Although no task was imposed on the distractors (only passive viewing), since distractors were shown at fixation at rapid succession, it was impossible to ignore them, as evidenced by the ceiling-level distractor decoding performance. Even with strong VWM representations present throughout OTC (including EVC) and PPC, when a linear classifier was trained on a pair of objects when they were distractors, to then decode the same objects when they were VWM targets during the same delay period, in both regions, a significant cross-decoding drop was obtained compared with within-decoding when training and testing were both performed within VWM targets during the delay. In V1–V4, cross-decoding did not differ from chance, indicating minimal overlap between perception and VWM in these brain areas. Given that visual representations in the sensory regions were much stronger for the distractors (visible and sensory) than for the VWM targets (invisible), if perception and VWM shared a representation, training on the distractors to decode the VWM targets should outperform within-decoding. The presence of a significant cross-decoding drop accompanied by chance-level cross-decoding in some sensory areas thus indicates distinctive perceptual and VWM representations, inconsistent with the sensory nature of VWM as put forward by the original proposal of the sensory account of VWM (see also Duan and Curtis, 2024, for a similar argument).

Meanwhile, a distractor-trained classifier was able to successfully decode targets during the encoding period with no cross-decoding drop in OTC (but not in PPC). Thus, similar representations were formed in sensory areas for perceptual representations, whether or not a stimulus is viewed actively during VWM encoding or passively during VWM delay. This provides further validation of the matched experimental settings for targets and distractors in the present experimental paradigm. Given PPC’s involvement in goal-directed visual processing (Xu, 2018a,b) and its ability to encode the same information differently based on task relevancy (Xu and Vaiziri-Pashkam, 2019; Taylor and Xu, 2024), it appears that PPC “transforms” input earlier in the process to differentiate targets from distractors. It may be argued that the representational difference between VWM and perception is due to the presence of distraction during the delay period, as Xu (2024) showed that target representations differed in different distractor conditions in OTC (but not in PPC). Given the ubiquitous presence of distractions in everyday vision (i.e., we do not usually stare at a blank screen when we hold information in VWM in the real world), distraction in VWM is the norm rather than the exception. The results from VWM under distraction would thus more closely resemble VWM in real-world vision than those without distraction. That being said, even in trials without distractions, significant representational change between perception and VWM was still observed across a number of ventral sensory areas, and there was no statistical evidence showing an effect of distraction here.

Assuming VWM representation during encoding is largely sensory in nature (especially in OTC), the same conclusion is also reached from cross-decoding results between VWM encoding and delay. Although prior work examined such cross-decoding (Riggall and Postle, 2012; Emrich et al., 2013; Sreenivasan et al., 2014; Kwak and Curtis, 2022; Xu, 2023), none included trials with distractors; additionally, some did not include PPC or explicitly quantify the difference between OTC and PPC. The present study found a significant cross-decoding drop across OTC and PPC, with the drop being greater in OTC than in PPC and unaffected by the presence of distractors. These results again showed a representational change between perception and VWM, indicating that representational transformation is a natural part of VWM consolidation and echoing the cross-decoding results obtained from training distractors to decode targets.

By training with a sensory task to decode the content of VWM, a previous study reported much lower perception to VWM cross-decoding in PPC than in EVC (Rademaker et al., 2019), leading to the conclusion that greater transformation between perception and VWM exists in PPC than in EVC. However, without participants attending to the stimuli in the sensory task and with PPC visual representations being more attention and task-driven than those in EVC (Xu, 2018a; see also a similar comment made by Rademaker et al., 2019), as noted by Xu (2020), the perceptual classifier employed in cross-decoding was likely much weaker in PPC than in EVC. This could have distorted the cross-decoding results in an unintended way. Without this complication, the present study found quite the opposite results, with PPC representations exhibiting less transformation and more stability and consistency across VWM processing than those in OTC. Meanwhile, because our study and Rademaker et al. (2019) differed in other aspects, such as stimuli and task demands, it remains a possibility that factors other than attention engagement could have contributed to the observed discrepancy in results.

The results of the present study thus support a transformed account of VWM representation in which perceptual input is transformed before it is stored in VWM, consistent with more recent evaluations of the sensory account of VWM (Adam et al., 2022). What could cause such a transformation? One possibility is that representations are naturally rotated between perception and VWM as part of the memory consolidation process, resulting in a significant cross-decoding drop. Indeed, rotation in visual representation has been observed in a host of other visual processes, including priority-based target coding in VWM (Xie, et al., 2022), separating attention-related versus VWM-related processing (Panichello and Buschman, 2021; Jahn et al., 2024), separating sensory input and stored long-term memory information (Libby and Buschman, 2021), separating target and distractor and separating target and target representations in VWM (Xu, 2024), and representing visual information in different tasks (Taylor and Xu, 2024).

Besides a rotation in the representational space, the transformation may also be caused by a change in the representational content between perception and VWM. At any given moment, only a fraction of the real-world perceptual input is relevant to behavior. A direct transfer of the perceived sensory information to VWM, as championed by the original proposal of the sensory account of VWM, is neither an efficient use of the limited VWM resources nor is it necessary. From the perspective of goal-directed visual information processing, significant transformations between perception and VWM are expected to allow the selection and consolidation of the most task-relevant information to be stored in VWM. Given PPC’s greater ability to encode task-relevant information than that of OTC during perception (Xu, 2018a) and PPC’s ability to drive the representational content of OTC during VWM delay (Xu, 2023), transformation is expected to be greater in OTC than in PPC. A task-driven transformed account of VWM can thus easily explain the present results and a host of recent findings showing transformed representations in VWM (Kwak and Curtis, 2022; Li and Curtis, 2023; Xu, 2023; Yan et al., 2023; Duan and Curtis, 2024). While it is possible to use tailor-made stimuli to elicit only the intended task-relevant features and then store them in VWM, thereby minimizing the transformation between perception and VWM to retain the sensory representations in VWM, such a scenario, however, is an exception, rather than the norm, of how visual information is normally extracted and stored in VWM, especially given the rich and complex nature of real-world visual inputs. The utility of the sensory nature of VWM representation as originally proposed by the sensory account of VWM is thus limited in this regard (see other drawbacks of this account, e.g., Bettencourt and Xu, 2016a; Leavitt et al., 2017; Xu, 2017, 2018c, 2020, 2021).

To conclude, the present study reevaluates critical evidence supporting the sensory nature of VWM representation in human sensory regions and shows that a task-driven transformed account better captures how visual information is retained in VWM in these regions.

## Data Availability

All data files and analysis scripts contributed to this study are available at https://osf.io/8rbkh/.

## Synthesis

Reviewing Editor: Alexander Soutschek, Ludwig-Maximilians-Universitat Munchen Decisions are customarily a result of the Reviewing Editor and the peer reviewers coming together and discussing their recommendations until a consensus is reached. When revisions are invited, a fact-based synthesis statement explaining their decision and outlining what is needed to prepare a revision will be listed below. The following reviewer(s) agreed to reveal their identity: Margaret Henderson. Note: If this manuscript was transferred from JNeurosci and a decision was made to accept the manuscript without peer review, a brief statement to this effect will instead be what is listed below.

The current study aims to test whether working memory and sensory representations share identical formats-an idea suggested in some early formulations of the sensory account (also known as the sensory recruitment model). The author shows a significant drop in cross-decoding performance between the working memory delay and distractor encoding,and between the working memory delay and working memory encoding. Although similar effects have been reported in previous studies (e.g., Rademaker et al., 2019; Kwak and Curtis, 2022), the current work provides stronger evidence by carefully matching conditions, as the author highlights. This supports the view that working memory representations are not a direct copy of sensory representations.

That said, I believe a few points could benefit from further clarification. One is how the sensory recruitment account is characterized. While some early studies (e.g., Serences et al., 2009; D'Esposito &Postle, 2015) described the account as involving both the engagement of sensory regions and a shared representational format between perception and working memory, more recent literature (e.g., Kirsten et al., Visual Memory, 2022; Yörük et al., Psychon. Bull. Rev., 2020) appears to focus on only the former, while acknowledging that working memory representations may transform during maintenance. Recent work emphasizes the engagement of sensory regions during WM maintenance, without necessarily assuming that WM codes are identical to sensory codes. Since the present study addresses the question of representational similarity, the conclusions may be more appropriately framed as a challenge to an earlier version of the account rather than testing a core prediction of its current form.

Another point. In the discussion, the author suggests that the difference in PPC decoding between the current study and Rademaker et al. (2019) may be due to differences in attentional engagement across tasks. While this is a reasonable possibility, the two studies also differ in other respects, including stimuli and task demands. It might be worth noting that attentional engagement is likely one of several potential factors contributing to the observed discrepancy.

## Author Response

I thank the editor and the reviewers for their time and effort, and their comments. I have addressed in detail below each comment raised by the new reviewer and have revised the manuscript accordingly. The changes made are marked in blue in the revised manuscript. For easy referencing, I include the reviewer's original comments in the italicized text below. ******************************** Reviewer comment :

The current study aims to test whether working memory and sensory representations share identical formats-an idea suggested in some early formulations of the sensory account (also known as the sensory recruitment model). The author shows a significant drop in cross-decoding performance between the working memory delay and distractor encoding, and between the working memory delay and working memory encoding. Although similar effects have been reported in previous studies (e.g., Rademaker et al., 2019; Kwak and Curtis, 2022), the current work provides stronger evidence by carefully matching conditions, as the author highlights. This supports the view that working memory representations are not a direct copy of sensory representations.

That said, I believe a few points could benefit from further clarification. One is how the sensory recruitment account is characterized. While some early studies (e.g., Serences et al., 2009; D'Esposito &Postle, 2015) described the account as involving both the engagement of sensory regions and a shared representational format between perception and working memory, more recent literature (e.g., Kirsten et al., Visual Memory, 2022; Yörük et al., Psychon. Bull. Rev., 2020) appears to focus on only the former, while acknowledging that working memory representations may transform during maintenance. Recent work emphasizes the engagement of sensory regions during WM maintenance, without necessarily assuming that WM codes are identical to sensory codes. Since the present study addresses the question of representational similarity, the conclusions may be more appropriately framed as a challenge to an earlier version of the account rather than testing a core prediction of its current form.

I thank the reviewer for raising this point. It is indeed the case that Adam et al. (2022, Visual Memory) focused on the involvement of the sensory areas in WM and discussed instances where perceptual and WM representations could differ. Although they did not emphasize the transformed nature of WM, their overall take on the sensory recruitment theory is consistent with the present manuscript. Yörük et al. (2020, Psychon. Bull.

Rev.) showed that task manipulation can modulate whether or not perception and WM share similar representational characteristics. This is again consistent with the present manuscript, showing that retaining a sensory-like representation in WM is not the norm and is highly task-dependent. Given that the earlier version of the sensory account is still highly cited today, much more so than the current version, I thought that it would be useful to make a more definitive evaluation in the present manuscript to state more explicitly what is still valid and invalid of the earlier version in light of new data. All in all, I agree that the field as a whole has reached a consensus on this topic. I have added texts throughout Abstract, Significance, and General Discussion (highlighted in blue) to reflect this discussion and have added these two citations (plus a third relevant citation mentioned in Yörük et al., 2020) to the revised manuscripts.

Another point. In the discussion, the author suggests that the difference in PPC decoding between the current study and Rademaker et al. (2019) may be due to differences in attentional engagement across tasks. While this is a reasonable possibility, the two studies also differ in other respects, including stimuli and task demands. It might be worth noting that attentional engagement is likely one of several potential factors contributing to the observed discrepancy.

This is a fair point, although Rademaker et al. (2019) also acknowledged that the lack of attentional engagement in their perceptual task could have contributed to their failure to obtain reliable sensory responses from PPC (p. 1342, 1st paragraph of the right column). I have added texts in General Discussion to reflect this comment (highlighted in blue).
