# Supplementary figures and images for: Transformed Visual Working Memory Representations in Human Occipitotemporal and Posterior Parietal Cortices
Source: eNeuro. 2025 Jul 8;12(7):ENEURO.0162-25.2025. doi: 10.1523/ENEURO.0162-25.2025 (PMC12243946; doi:10.1523/ENEURO.0162-25.2025)

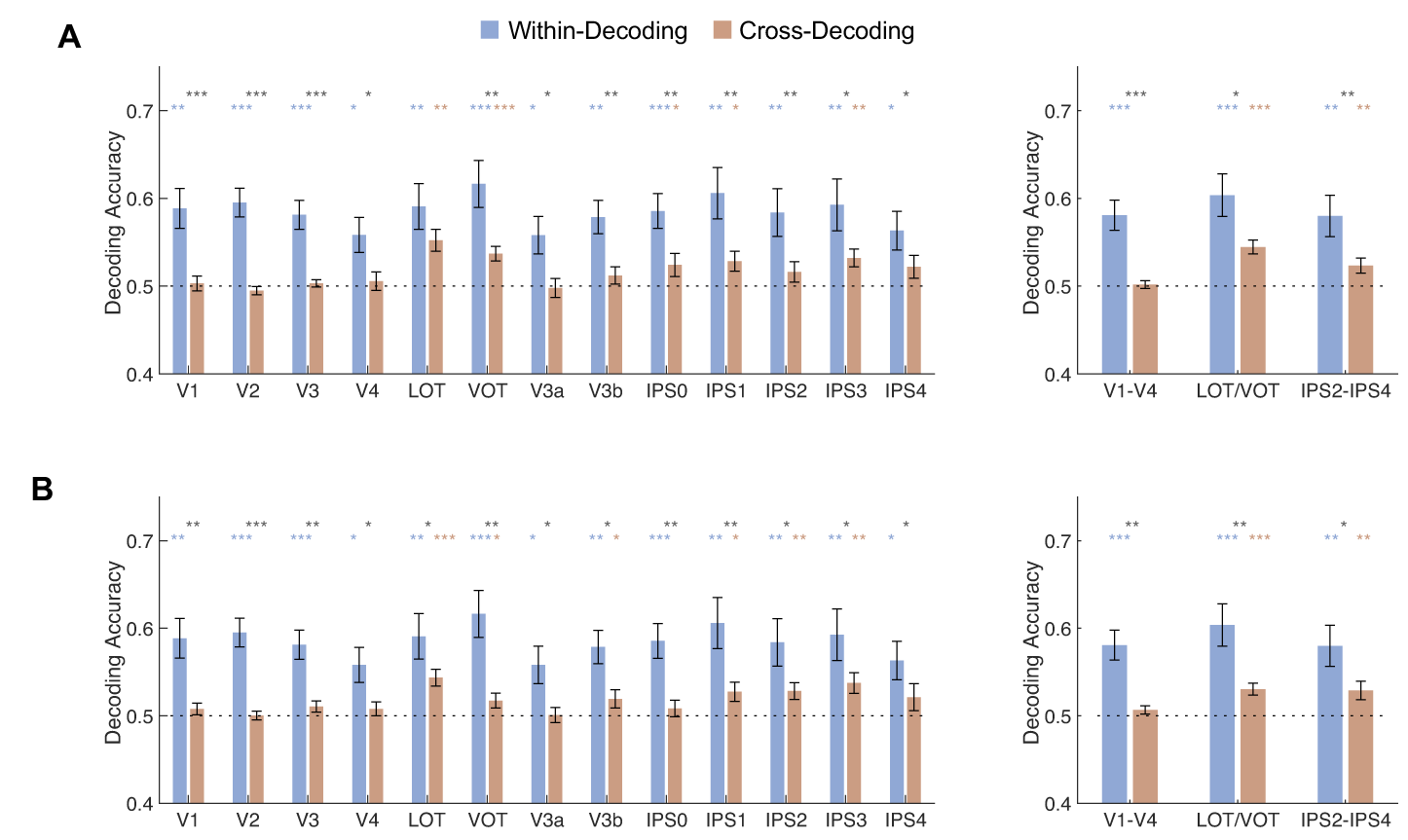

Supplement: Figure 2-1 — Training distractors to decode targets in trials with distractors. A. The irrelevant object was the same across training and test (e.g., training on A vs. B when they were distractors with C being the target in both to decode A vs. B when they were targets with C being the distractor in both). B. The irrelevant object differed across training and test (e.g., training on A vs. B when they were distractors with C being the target in both to decode A vs. B when they were targets with D being the distractor in both). Error bars indicate s.e. * p < .05, ** .01 < p < .001, *** p < .001. Download Figure 2-1, TIF file. [file eneuro-12-ENEURO.0162-25.2025-s001.tif]
